# Further Characterisation of the Molecular Signature of Quiescent and Activated Mouse Muscle Satellite Cells

**DOI:** 10.1371/journal.pone.0005205

**Published:** 2009-04-16

**Authors:** Viola F. Gnocchi, Robert B. White, Yusuke Ono, Juliet A. Ellis, Peter S. Zammit

**Affiliations:** Randall Division of Cell and Molecular Biophysics, King's College London, Guy's Campus, London, United Kingdom; McMaster University, Canada

## Abstract

Satellite cells are the resident stem cells of adult skeletal muscle. To date though, there is a paucity of native markers that can be used to easily identify quiescent satellite cells, with Pax7 probably being the best that is currently available. Here we have further characterized a number of recently described satellite cell markers, and also describe novel ones. Caveolin-1, integrin α7 and the calcitonin receptor proved reliable markers for quiescent satellite cells, being expressed by all satellite cells identified with Pax7. These three markers remained expressed as satellite cells were activated and underwent proliferation. The nuclear envelope proteins lamin A/C and emerin, mutations in which underlie Emery-Dreifuss muscular dystrophy, were also expressed in both quiescent and proliferating satellite cells. Conversely, Jagged-1, a Notch ligand, was not expressed in quiescent satellite cells but was induced upon activation. These findings further contribute to defining the molecular signature of muscle satellite cells.

## Introduction

The satellite cell is the resident stem cell of growing and adult skeletal muscle, located between the plasmalemma and the surrounding basal lamina of a muscle fibre [1]. During adult life there is low myonuclear turnover, with only a sporadic requirement for hypertrophy or repair, so satellite cells become mitotically quiescent. When new myonuclei are required, satellite cells are activated to proliferate and differentiate, either fusing to existing myofibres or fusing together to form new myotubes [2]. Despite accounting for only between 1–4% of the total myofibre nuclei [3], satellite cells are able to fully regenerate a muscle in only a matter of days following total destruction using myotoxins [4], [5]. Importantly, satellite cells are able to self-renew, as shown by grafting experiments, where a single myofibre (with a mean of ∼7 satellite cells) is able to give rise to both many new myonuclei and satellite cells [6], so maintaining a viable stem cell pool throughout life.

Besides the criterion of their specific anatomical localization, quiescent satellite cells can also be identified by the expression of a peculiar set of molecular markers. The most widely used in mouse are probably the paired-box transcription factor Pax7 [7], M-cadherin [8] and CD34 [9]. Recently we have shown that satellite cells also have high levels of sphingomyelin in their plasma membranes, and this sphingolipid can be detected using the protein lysenin [10]. Other reported markers of quiescent satellite cells now include the heparin sulphate proteoglycans syndecan 3 and 4 [11], FoxK1 (formerly myocyte nuclear factor) [12], Sox 8 [13], Sox 15 [14] and the antibody SM/C2.6 [15]. In addition, there are various genetically modified mice that provide a means to identify satellite cells, including the products of the targeted alleles in *Myf5^nlacZ/+^* [9] and *Pax3^eGFP/+^* [16] mice, and a nestin transgene [17]. Once activated, satellite cells rapidly induce expression of the basic helix-loop-helix transcription factor MyoD, together with other muscle specific genes, and markers common to proliferating cells in general [18].

Interestingly, the expression profiles in some genetically modified mice have indicated that there may be heterogeneity within the satellite cell compartment [16], [19], and so it is important to determine whether it is possible to use the native molecular signature to distinguish between satellite cell populations. Here, we have used several recently described markers of quiescent satellite cells to determine whether they recognize all satellite cells. Markers in the plasma membrane also have the advantage that they can potentially be useful for FAC sorting to obtain defined cell populations. To this end, we examined expression of caveolin-1, integrin α7, calcitonin receptor (CTR), emerin, lamin A/C and jagged-1 in quiescent and activated satellite cells.

Caveolin-1 is a principal component of caveolae that has recently been proposed as specifically expressed in quiescent satellite cells [20], in contrast to the muscle-specific caveolin-3 expressed at the sarcolemma, mutation in which causes LGMD1C [21]. Mutation in integrin α7 has been shown to underlie a congenital myopathy [22] and is present on quiescent satellite cells [23], [24]. The CTR is present on quiescent satellite cells and calcitonin delays activation [25]. In addition we also examined the expression of nuclear envelope proteins lamin A/C and emerin, mutations in which cause autosomal-Emery-Dreifuss muscular dystrophy (A-EDMD) [26] and X-EDMD [27] respectively, since there is evidence that these mutations may directly affect satellite cell function [reviewed in 28]. By immunostaining isolated myofibres, we were able to examine the entire satellite cell population, and precisely determine on a cell-by-cell basis, the percentage of satellite cells *in situ* that actually express the given marker. We found caveolin-1, integrin α7, CTR, lamin A/C and emerin to all be good markers of quiescent and activated satellite cells from various muscles. Finally, Notch signaling is involved in control of satellite cell activation and proliferation [40], and we found that the Notch ligand Jagged-1 [29] was induced during satellite cell activation.

## Materials and Methods

### Myofibre isolation

Mice were bred, and experimental procedures were carried out, in accordance with British law under the provisions of the Animals (Scientific Procedures) Act 1986. Male C57 Bl/10 mice aged 8–12 weeks were killed by cervical dislocation and the extensor digitorum longus (EDL), soleus and masseter muscles were carefully dissected. Muscles were digested in 0.2% Collagenase Type 1/DMEM (Sigma); individual myofibres were dissociated by trituration and washed, as described in detail elsewhere [30]. Careful microscopic examination was then performed to ensure that selected myofibres did not have any capillaries still associated [31]. Myofibres were fixed for 10 minutes in 4% paraformaldehyde/PBS (Sigma) within 2 hrs of mouse sacrifice, in order to maintain the associated satellite cells as near to quiescence as possible.

### Myofibre culture

To induce satellite cell activation, myofibres were cultured in suspension in DMEM (Gibco) containing 10% (v/v) horse serum (Gibco), 0.5% (v/v) chick embryo extract (MP Biomedicals Europe), 4mM L-Glutamine (Sigma) and 1% (v/v) penicillin/streptomycin solution (Sigma) at 37°C in 5% CO_2_. Myofibres were then fixed with 4% paraformaldehyde/PBS at the desired time points.

### Semi-quantitative RT-PCR

Myofibres were stripped of satellite cells either immediately upon isolation, or following 48 hrs in culture, by digestion in 0.125% Trypsin/EDTA at 37°C for 15 mins followed by gentle trituration. Myofibre fragments were removed by passing through a 40 µm cell strainer (BD Falcon), and satellite cells collected by centrifugation, washed twice with PBS, and total RNA was isolated (RNeasy; Qiagen) and reverse transcribed using the Quanti-Tect kit (Qiagen). Cycling parameters were 94°C/20 s, 58°C/20 s, 72°C/20 s for 32–36 cycles and PCR products were resolved on 1.5% agarose gels. PCR was conducted with the following primers, designed using Primer-BLAST (NCBI):

*Caveolin-1*: F - GCACACCAAGGAGATTGACC; R - GAATGGCAAAGTAAATGCCC;

*Integrin-α7*: F - CAATCTGGATGTGATGGGTG; R - CTCAGGGGACAAGCAAAGAG;

*CTR*: F - CTGCTGCTCCTGCTCCTAGT; R - AGTGCTGATAGGCTGTGGCT;

*Emd*: F - GGACGACTATGCGGTTTTGT; R - TACATGTCTGAGTCCACGGC;

*Lmna*: F - AGGACCTCGAGGCTCTTCTC; R - CTCCTTCAGCGTCTGTAGCC;

*Jag-1*: F - CCCCCTGAGTCTTCTGCTC; R - GTGACGCGGGACTGATACTC;

*Pax7*: F - CCGTGTTTCTCATGGTTGTG; R - GAGCACTCGGCTAATCGAAC;

*MyoD*: F - AGCACTACAGTGGCGACTCA; R - GCTCCACTATGCTGGACAGG;

*Myogenin*: F - CTACAGGCCTTGCTCAGCTC; R - AGATTGTGGGCGTCTGTAGG;

*Gapdh*: F - GTGAAGGTCGGTGTGAACG; R - ATTTGATGTTAGTGGGGTCTCG.

### Immunocytochemistry

Primary antibodies used were: mouse monoclonal anti-Pax7 (Developmental Studies Hybridoma Bank); rabbit polyclonal anti-caveolin-1 (N20) (Santa Cruz Biotechnology); rabbit polyclonal anti-calcitonin receptor (AbD Serotec); mouse monoclonal anti-MyoD clone 5.8A (DakoCytomation); rabbit polyclonal anti-MyoD (Santa Cruz Biotechnology); goat polyclonal anti-Jagged-1 (C20) (Santa Cruz Biotechnology); mouse monoclonal anti-lamin A/C (131C3) (GeneTex Inc.); rabbit polyclonal anti-integrin α7 (U31) [32] and rabbit polyclonal anti-emerin (AP8), raised against human emerin residues 1–70 [33].

Immunostaining was performed as described in detail elsewhere [9]. Briefly, the paraformaldehyde-fixed myofibres were permeabilised with 0.5% (v/v) Triton X-100 in PBS for 6 mins and then blocked using 10% (v/v) goat serum (DakoCytomation) and 10% (v/v) swine serum (DakoCytomation). 10% horse serum replaced goat serum when a primary antibody raised in goat was used. Primary antibodies were usually applied overnight at 4°C and the myofibres then washed in PBS/0.025% Tween 20. Species-specific fluorochrome-conjugated secondary antibodies (Invitrogen) were then applied for 1–2 hrs, before further washing and mounting in Vectashield mounting medium (Vector Laboratories, Inc.) containing 100 ng/ml 4,6-diamidino-2-phenylindole (DAPI).

### Microscopy

The images were acquired using either a LSM 5 *EXCITER* confocal microscope equipped with a water immersion LD C-Apochromat 40×/1.1 W Corr objective (Zeiss) with acquisition software ZEN 2007 LSM (Zeiss), or a Zeiss Axiophot 200M microscope with a Charge-Coupled Device (Zeiss AxioCam HRm) using AxioVision software version 4.4 (Zeiss). Figures were processed and assembled into figures using Adobe Photoshop CS.

## Results

**Figure 1:**
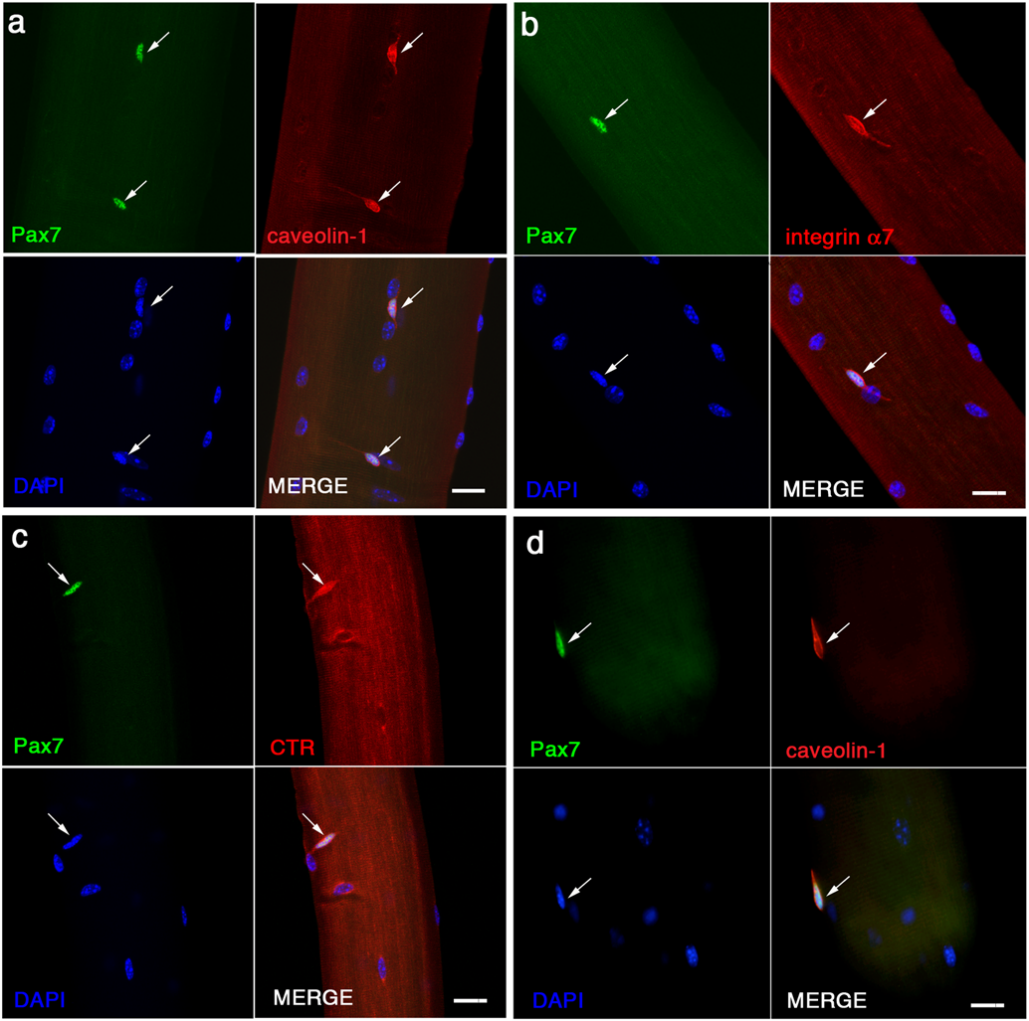
Caveolin-1, integrin α7 and CTR are expressed by Pax7+ve quiescent satellite cells in their niche on the myofibre. Satellite cells in their niche on the surface of freshly isolated EDL myofibres were co-immunostained for Pax7 (green) and either caveolin-1 (a - red), integrin α7 (b - red) or CTR (c - red). All three proteins were expressed in the plasma membrane of Pax7+ve satellite cells (arrowed), clearly revealing their bi-polar morphology. Myofibres were also examined from the masseter of the jaw, and again Pax7+ve satellite cells expressed all three markers (caveolin-1 shown as an example in d). All nuclei were counterstained with DAPI (blue), revealing the presence of the myonuclei of the myofibres on which the satellite cells were located. Scale bar represents 20 µm.

The main purpose of this study was to further characterise the expression profile of a number of reported and novel markers of satellite cells. To do this we isolated myofibres from the muscles of adult (8–12 week-old) mice and then immunostained the associated satellite cells. Myofibres were isolated and fixed as soon after isolation as possible (∼2 hrs from mouse sacrifice). The time taken for their isolation means that the activation process is probably initiated, but satellite cells prepared in this way are mainly MyoD-negative, an early molecular landmark in activation [9], [18]. This procedure therefore, allows examination of near-quiescent satellite cells retained in their niche on the surface of the myofibre. In the absence of other clearly defined molecular stages in early activation though, we henceforth refer to MyoD-negative satellite cells on freshly isolated myofibres as “quiescent”. We have previously shown that Pax7 recognizes the entire satellite cell population in young mice [34], so we co-immunostained for Pax7 and the antibody of interest (Figure 1). This technique thus allows us to determine the percentage of the satellite cell population that is recognised by a particular marker.

### Caveolin-1, integrin α7 and CTR are good cell surface markers for quiescent satellite cells

Caveolin-1, integrin α7 and CTR were robustly expressed in the plasma membranes of quiescent satellite cells identified by Pax7 expression (Figure 1). The characteristic morphology of a quiescent satellite cell as a bi-polar cell with long processes is evident using these surface markers. Immunostaining for MyoD revealed that all the Pax7 positive (+ve) satellite cells had not progressed to the point of inducing *MyoD* expression, so can be considered as quiescent (data not shown). Using Pax7 expression as a denominator, we found that practically all EDL satellite cells express caveolin-1, integrin α7 and CTR in their plasma membranes (Table 1). The CTR immunosignal was generally weaker than those for caveolin-1 and integrin α7, and so we feel that the very rare cells that were negative for CTR probably result from signal below the level of detection, rather than comprising a separate marker-negative population.

**Table 1:** Percentage of quiescent satellite cells expressing each marker

	Percentage of Pax7+ve satellite cells with the marker
**Caveolin-1**	**100±0** (n = 493)
**Integrin α7**	**99.6±0.3** (n = 248)
**Calcitonin receptor**	**98.5±0.6** (n = 282)

Mean±SEM from at least 15 EDL myofibres from each of at least 3 mice.

The EDL is mainly composed of fast type IIx and IIb fibre types [35], but Pax7+ve satellite cells were also recognized by caveolin-1, integrin α7 and CTR on myofibres from the soleus (data not shown), which is composed of slow type I and fast IIa fibre types in mouse. The EDL and soleus are derived from cells that migrate from the somites during development and so we also examined the expression of these markers in the masseter of the head, which is derived from paraxial head and pre-cordal mesoderm [reviewed in 36]. Again, we found that Pax7+ve satellite cells in masseter were also recognized by caveolin-1 (Figure 1d), integrin α7 and CTR (data not shown).

### Lamin A/C and emerin are localized around the nuclei of Pax7+ve quiescent satellite cells

**Figure 2:**
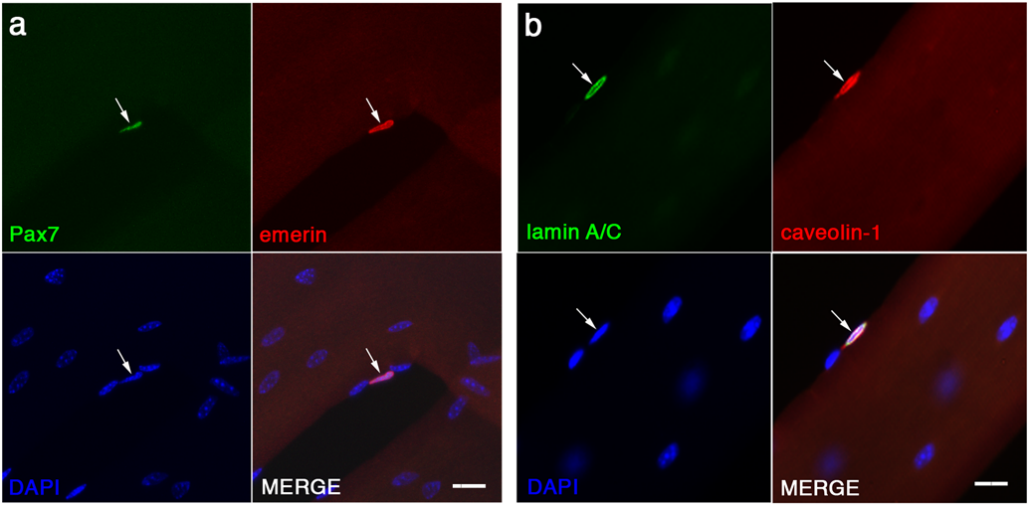
Emerin and lamin A/C are expressed in the nuclear envelope of quiescent satellite cells. Freshly isolated EDL myofibres were co-immunostained for Pax7 and emerin (a). Emerin (red) was expressed in the nuclear envelope of Pax7+ve (green) satellite cells (arrow). Since a mouse monoclonal antibody was used to immunostain lamin A/C, satellite cells were identified using caveolin-1 (b). We found that the nuclear lamina of caveolin-1 (red)-expressing satellite cells (arrow) contained lamin A/C (green). All nuclei present were revealed by counterstaining with DAPI (blue). Scale bar represents 20 µm.

Mutations in *LMNA* and *EMD* that underlie A-EDMD and X-EDMD respectively, are speculated to also perturb satellite cell function, so directly contributing to disease progression [reviewed in 28]. Thus, we were interested in investigating and characterizing lamin A/C and emerin expression in satellite cells. Emerin, encoded by the *EMD* gene, is an integral membrane protein with cell-type specific locations, which in striated muscle, include the inner and outer nuclear membrane, nucleoplasm and sarcoplasmic reticulum [reviewed in 37]). Co-immunostaining of Pax7 with emerin revealed a perinuclear signal, clearly showing that quiescent satellite cells express emerin at their nuclear envelope, with some signal also evident in the nucleoplasm (Figure 2a).

Nuclear lamins are type V intermediate filaments and are the principal component of the nuclear lamina, a complex protein meshwork underlying the nuclear envelope [38]. Lamins A and C are the principal A-type lamins and are encoded by alternative splicing from the *LMNA* gene. Since we used a monoclonal antibody that recognizes both lamin A and C, we could not co-immunostain for Pax7. Instead we co-immunostained satellite cells for lamin A/C and caveolin-1. Again we detected a clear perinuclear signal, demonstrating that lamin A/C is present in the nuclear lamina of quiescent satellite cells (Figure 2b). It should be noted however, that the nuclear envelope of myonuclei also contain emerin and lamin A/C, but our immunostaining procedure is optimised to examine satellite cells.

### Caveolin-1, integrin α7, CTR, emerin and lamin A/C are present in activated satellite cells

Although mitotically quiescent for much of the time in adult muscle, satellite cells are activated to proliferate and differentiate to provide new myonuclei to meet the routine needs of muscle homeostasis, or the more sporadic demands for hypertrophy or repair. An early step in this activation process is the induction of *MyoD* expression. These early stages of satellite cell activation can be modeled *in vitro* by culturing isolated myofibres in medium containing horse serum and chick embryo extract. The vast majority of associated satellite cells in their niche co-express Pax7 with MyoD within hours of mitogen exposure [39] and re-enter the cell cycle, undergoing mitosis between ∼30–40 hrs.

**Figure 3:**
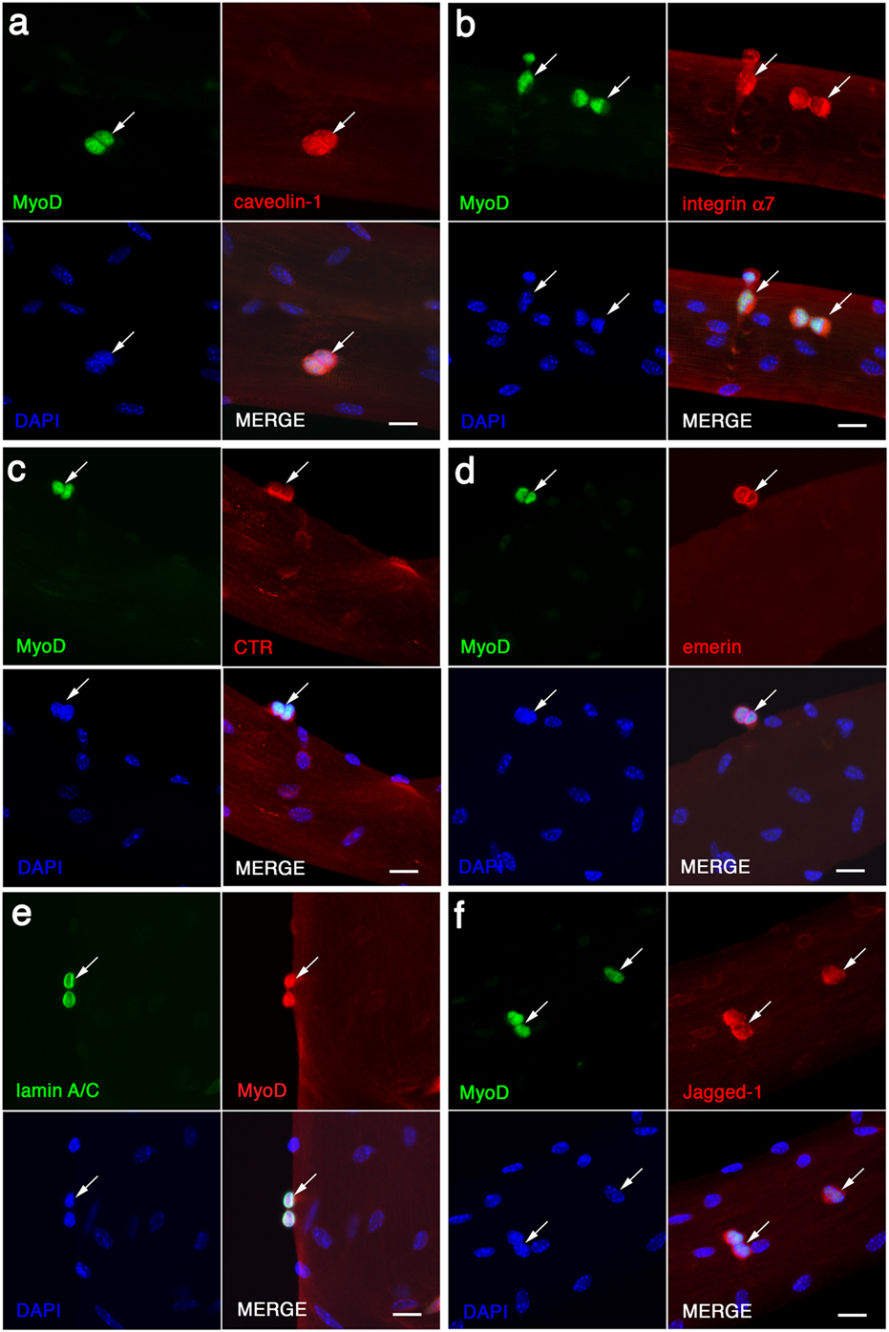
Jagged-1 is induced when satellite cells are activated. EDL myofibres were cultured in suspension for 48 hrs in plating medium to elicit the activation and proliferation of satellite cells (arrows). Activated and proliferating satellite cells were identified by immunostaining for MyoD (green), and Caveolin-1 (a - red), integrin α7 (b - red) and CTR (c - red) remained expressed in the plasma membrane of activated satellite cells. The continued presence of both emerin (red, d) and lamin A/C (green, e) in the nuclear envelope of activated satellite cells expressing MyoD (green in d, red in e) was also noted. Moreover, we found that the Notch ligand Jagged-1 (red) was induced upon activation, in MyoD+ve (green) satellite cells (f). Nuclei were counterstained with DAPI (blue). Scale bar represents 20 µm.

To examine the expression profiles of caveolin-1, integrin α7, CTR, emerin and lamin A/C, isolated myofibres were cultured for 48 hrs, before being fixed and immunostained for MyoD and the relevant marker (Figure 3). Caveolin-1 (Figure 3a), integrin α7 (Figure 3b) and CTR (Figure 3c) remained detectable on the surface of MyoD+ve activated and proliferating satellite cells. As in quiescent satellite cells, all three markers were found at the cell membrane and revealed the shape of the cell at this stage of activation: activated and proliferating satellite cells have a rounder morphology than when quiescent. Satellite cells were often seen as doublets at this time, presumably the progeny of a recent cell division.

Emerin and lamin A/C remained nuclear associated in MyoD+ve activated and proliferating satellite cells (Figure 3d and e) after 48 hrs in culture. The nuclear envelope is reorganized during mitosis and so the utility of these markers is dependent of the specific phase of the cell cycle that the satellite cell is in when immunostained.

### Jagged-1 expression in satellite cells is induced upon activation

The markers examined to date were expressed in quiescent satellite cells, and the proteins remained present as the satellite cells are activated and begin to proliferate. Activated and proliferating satellite cells also induce a whole cohort of markers universal to dividing cells, together with those specific to myoblasts. We examined several other markers and of particular interest was Jagged-1, a single pass transmembrane Notch ligand in vertebrates [29]. Notch signaling is involved in activation of satellite cells [40]. It has been shown that activated Notch-1 appears during satellite cell activation, promoting myogenic precursor expansion [40]. Jagged-1 was not expressed in quiescent satellite cells in their niche on the myofibre (data not shown), but its expression was induced during activation, so that by 48 hrs in culture, many MyoD+ve satellite cells contained Jagged-1 (Figure 3f).

### The mRNA levels of the analyzed markers reflect the protein levels

**Figure 4:**
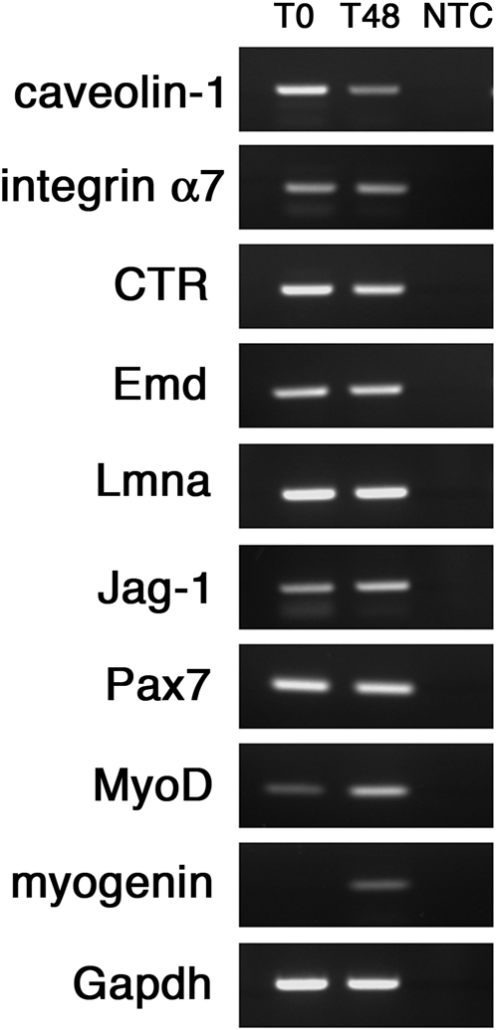
Marker protein levels reflect gene expression in quiescent and activated satellite cells. Total RNA was obtained from quiescent (T0) and activated (T48) satellite cells and transcript-specific primers used to semi-quantitatively amplify the mRNA of markers. Amplification of Gapdh transcript was used as control of the RNA content. Pax7, MyoD and myogenin transcripts levels were included as control of the activation progression. While *caveolin-1* and *CTR* transcripts appeared to be more abundant in satellite cells at T0, mRNA for *integrin α7*, *Emd* and *Lmna* were unaltered. *Jagged-1* and *MyoD* transcript were increased in activated satellite cells, reflecting the protein levels. *Myogenin* transcript, as expected, was absent in quiescent satellite cells and appeared after 48 hrs in culture. NTC is no-template control.

Having established the dynamics of the different marker proteins in quiescent and activated satellite cells using immunocytochemistry, we wished to compare the mRNA expression profiles of these genes, so we performed semi-quantitative RT-PCR of their transcripts. We collected quiescent satellite cells (T0), and activated satellite cells after 48 hrs in culture (T48) from isolated myofibres, and purified the RNA. Amplifying the transcripts of the different markers we found that the mRNA levels were in accordance with the protein levels observed using immunostaining (Figure 4). *Caveolin-1, integrin α7, CTR, Emd* and *Lmna* transcripts were present in both quiescent and activated satellite cells, with a slightly higher amount in quiescent satellite cells compared to activated ones for *caveolin-1* and *CTR*. Interestingly, the *Jagged-1* transcript was already present at T0 - although we could not detect Jagged-1 protein by immunocytochemistry - but was more abundant in activated satellite cells. As expected, the mRNA levels of *MyoD* increased upon activation.

### Caveolin-1 is down regulated in differentiating satellite cells

**Figure 5:**
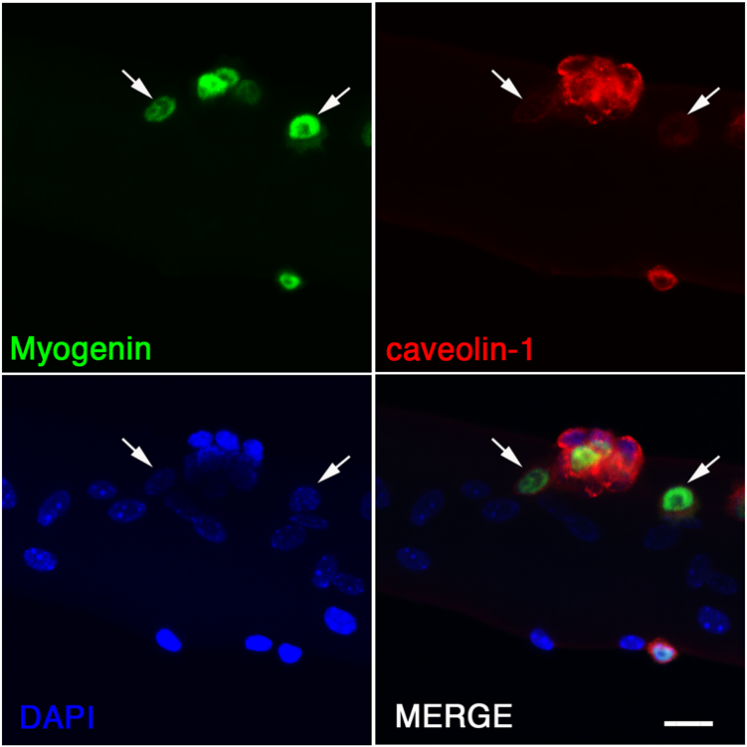
Caveolin-1 is down-regulated in differentiating satellite cell progeny. EDL myofibres were cultured in suspension for 72 hrs, by which time many begin to express myogenin as they commit to myogenic differentiation (arrows). Myogenin-containing satellite cells (green – arrowed) tended to have lower levels of caveolin-1 immunosignal (red - arrow) in their plasma membranes than uncommitted cells. Nuclei were revealed by counterstaining with DAPI (blue). Scale bar represents 20 µm.

Satellite cells maintained in culture for extended periods can either differentiate, apoptose or return to a quiescent-like state [39]. We have previously shown that levels of sphingomyelin in the plasma membrane of satellite cells with the self-renewal phenotype again increase to levels seen in quiescent cells [10], and a nestin transgene that is highly expressed in quiescent satellite cells is also re-induced in self-renewing cells [17]. We examined levels of caveolin-1 in satellite cells after 72 hrs of culture, when these fate choices are being made, and found that caveolin-1 immunostaining was stronger in the cells not containing myogenin, and so not yet udergoing myogenic differentiation (Figure 5).

## Discussion

Satellite cells are the resident stem cells of growing and adult skeletal muscle and represent a promising resource for the development of cell-based therapies for a number of muscle diseases. Thus, it is of importance to define a specific molecular signature for satellite cells, in order to identify and manipulate them efficiently.

To date, probably the most reliable markers to identify most mouse satellite cells is either immunostaining for Pax7 [7], or detecting β-gal from the targeted allele in the *Myf5^nlacz/+^* mouse [9]. Plasma membrane markers including CD34, SM/C2.6 and integrin α7 have all been used for obtaining satellite cells by FAC sorting [23], [25]. Here we examined the expression profile of other reported plasma membrane markers of satellite cells, and found that caveolin-1, integrin α7 and CTR are expressed by quiescent Pax7+ve satellite cells in their niche on myofibres from EDL, soleus and masseter muscles. Both the rabbit polyclonal antibodies against caveolin-1 and CTR are commercially available and so are accessible tools to identify satellite cells by immunocytochemistry [20], [25].

The use of genetically modified mouse models including the *Pax3^eGFP/+^* and *Myf5^Cre/+^* targeted alleles has raised the possibility that the satellite cell population may be heterogeneous [16], [19]. Careful quantification of the number of Pax7-containing satellite cells that co-expressed these markers on EDL myofibres showed that caveolin-1 and integrin α7 were present in all quiescent satellite cells. For CTR the figure was ∼98.5%, with the rare cells that were classified as negative probably a result of the lower immunosignal with this antibody, rather than the presence of a permanent sub-population. Therefore, these markers further contribute to a molecular signature common to all satellite cells.

Both caveolin-1 and CTR have been reported to be significantly down-regulated in activated/proliferating satellite cells, with the protein no longer detectable [20], [25], while expression of integrin α7 has not been thoroughly examined. Myofibres cultured in mitogen-rich medium results in activation of the associated satellite cells, which then begin to divide after ∼30–40 hrs. We found that message levels of caveolin-1 and CTR were indeed slightly lower in activated and proliferating satellite cells, but their proteins remained readily detectable in the plasma membrane of MyoD+ve cells. Integrin α7 was also strongly expressed in activated satellite cells, which was reflected in the presence of the protein on the cell surface. Later in culture, satellite cells either differentiate, apoptose or adopt a phenotype consistent with self-renewal [39] and at this time, caveolin-1 was associated more with satellite cells that were not expressing myogenin, and so not undergoing myogenic differentiation. Integrin α7 and CTR also appeared to be down-regulated as satellite cell differentiated.

In addition to caveolin-1, integrin α7 and CTR, we also examined the expression profile and domain of both lamin A/C and emerin in satellite cells. There is evidence that satellite cell function is affected in EDMD, which may directly contribute to disease progression [reviewed in 28]. A hallmark of muscle in EDMD is heterochromatin condensation and convoluted nuclear membranes, leading to an increase in nuclear fragility and downstream muscle-specific gene deregulation. Interestingly, it has recently been reported that the nuclear membranes of satellite cells from EDMD patients are structurally intact [41]. In many cell types, including myocytes, emerin is located to both the nuclear membrane and nucleoplasm [37]. Here we show a similar location in both quiescent and activated satellite cells, suggesting this is a common position for emerin in muscle cells. Unlike emerin though, lamin A/C locates exclusively to the peri-nuclear region and nucleoplasm in all cell types studied, and we can now confirm this same localization in satellite cells. It is thus not clear why the nuclear envelope of satellite cells retains a normal structure in EDMD [41], when it contains mutant lamin A/C proteins or lacks emerin.

Notch signaling has been shown to be important in the activation and control of satellite cell proliferation [40]. Notch 1, 2 and 3 are expressed in satellite cells [25], [40], but less is known of the expression profiles of their ligands; delta-like 1, 3 and 4, and Jagged-1 and -2 in mammals. The availability of a good commercial antibody to Jagged-1, prompted us to examine its expression profile. We found that Jagged-1 was not expressed in Pax7+ve quiescent satellite cells, but was up-regulated during activation, being robustly expressed by MyoD+ve activated and proliferating satellite cells, in accordance with the appearance of activated Notch-1 [40].

Caveolin-1, integrin α7 and the calcitonin receptor therefore, provide good candidates for identifying quiescent and activated mouse satellite cells that can now be analyzed for their utility in man. To date, only a small number of surface markers are available to identify and isolate human satellite cells, including CD56 (N-CAM), M-cadherin, Syndecan-4, c-Met and CD34 [42]–[45]. Defining an accurate gene expression profile for satellite cells will not only be central to understanding their function, but also to manipulating the satellite cell pool as part of possible therapeutic interventions.
